# Supplementary material for: Progress in affinity ligand-functionalized bacterial magnetosome nanoparticles for bio-immunomagnetic separation of HBsAg protein
Source: PLoS One. 2022 Jul 25;17(7):e0267206. doi: 10.1371/journal.pone.0267206 (PMC9312401; doi:10.1371/journal.pone.0267206)
Supplement: S1 Raw images — (PDF) [file pone.0267206.s002.pdf]

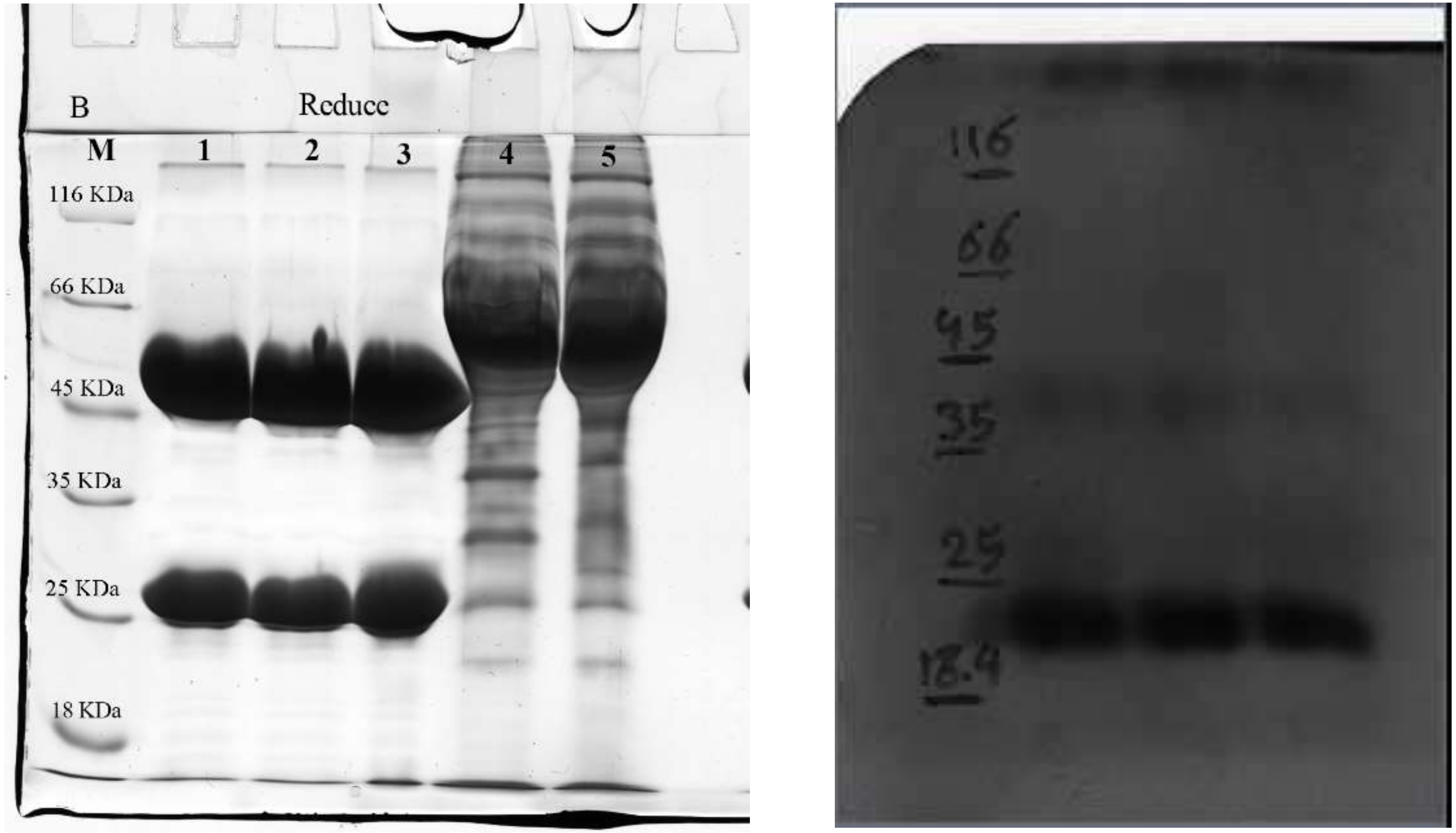

Fig. 3: (C) Coomassie Blue stained SDS PAGE (12%) of the purified Mab in reducing form: M (Ladder 302 KD), lanes 1, 2, and 3 (Purified Monoclonal antibody (Mab) or (eluted protein from protein G 303 column), lanes 4 and 5 (ascites fluid).

(D) Western blot analysis of the r-HBsAg exposed to mAb 304 of P1C7 hybridoma cell line, M (Ladder KD), lane 1, 2, and 3 (pure r-HBsAg) after developed 305 with purified Mab.

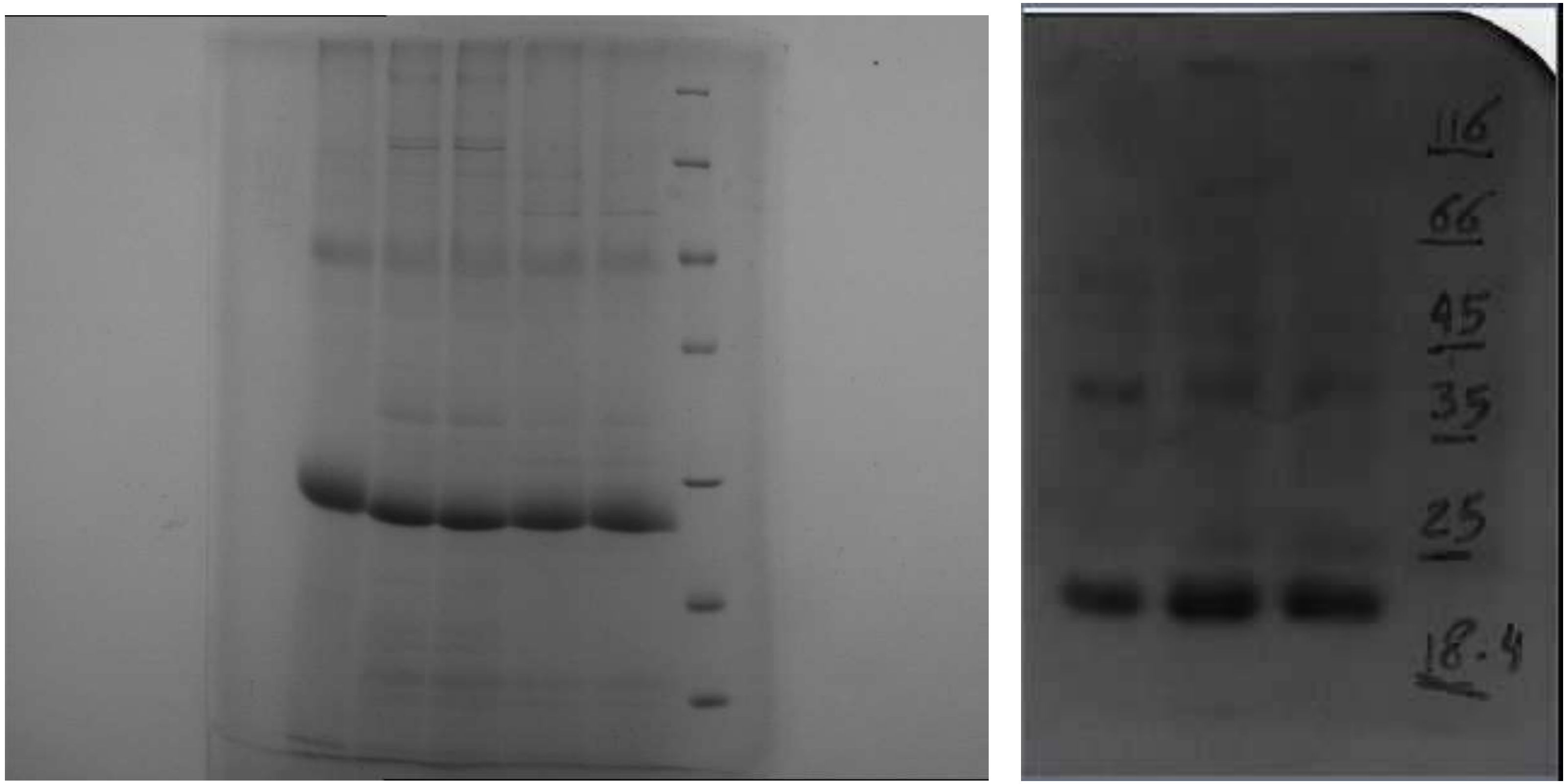

Fig 5. Characterization of eluted rHBsAg by immunomagnetic separation (IMS) or immunoaffinity chromatography (IAC). (A) SDS-PAGE analysis show the 24 KD band corresponding to rHBsAg purified by immunomagnetic separation method. (B) Western blot analysis show the 24 KD band corresponding to rHBsAg purified by immunomagnetic separation method.
